# Marital satisfaction among Tunisian women victims of domestic violence

**DOI:** 10.1192/j.eurpsy.2023.2405

**Published:** 2023-07-19

**Authors:** R. Jbir, L. Aribi, I. Chaari, M. Turki, N. Messedi, J. Aloulou

**Affiliations:** Hedi Chaker university hospital, Sfax, Tunisia

## Abstract

**Introduction:**

The phenomenon of domestic violence poses, long been a problem at the social and family. The magnitude of the consequences, associated with this issue justifies the importance placed on the quality of marital satisfaction among abused women.

**Objectives:**

To study the quality of marital satisfaction among women victims of domestic violence and to determine predictors of bad marital satisfaction

**Methods:**

Our study was descriptive and analytical cross-sectional, carried out with women examined in the context of medical expertise at ‘Hedi Chaker hospital’,Sfax , from May 2021 until January 2022.

An anonymous survey was asked to these ladies.

The AZRIN questionnaire was used to study the quality of marital satisfaction

**Results:**

The age oscillates between 18 and 64 years.

The half of the population (51.6%) had an average socio-economic level.

43.4% (n=53) lived in rented houses, 41% (n=50) owned the houses, 14.8% (n=18) lived with the husband’s family and 0.8% (n=1) was homeless.

All the women of our population were married: it was the first marriage in (89.3%).

The majority (86.1%) had children.

The average duration of marriage in our study was 11.16 years ± 9.12 years (min=1,max=40).

66.4% (n=81) were abused by their spouses during the first year of marriage.

Forty-seven women (38.5%) lived this ordeal daily.

98.7%were victim of verbal violence,94.7% of physical violence, 97.3% of psychological violence and 54.7 %of sexual violence.

Marital satisfaction was poor among 71,3% of ladies , average among 9% and good among 19,7%.

Bad marital satisfaction was significantly correlated with verbal violence (p=0,02),physical violence (p=0,01),psychological violence (p= 0,003)and sexual violence (p=0,04).

**Conclusions:**

As our results have shown, we do not expect assaulted women to be satisfied in their couple relationships.

Urgent help must be provided to these women to save them from this burden.

**Disclosure of Interest:**

None Declared

